# Programmed Cell Death-1/Programmed Death-Ligand 1 Blockade Improves Survival of Animals with Sepsis: A Systematic Review and Meta-Analysis

**DOI:** 10.1155/2018/1969474

**Published:** 2018-08-12

**Authors:** Qiang Zhang, Zhijiang Qi, Bo-Liu, Chun-Sheng Li

**Affiliations:** Beijing Key Laboratory of Cardiopulmonary Cerebral Resuscitation, Beijing Chao-Yang Hospital, Capital Medical University, Beijing 100020, China

## Abstract

**Object:**

To investigate effects of programmed cell death-1 (PD-1) related blockade in sepsis animals.

**Methods:**

Two reviewers independently searched electronic databases including PubMed, EMBASE, and the Cochrane Library up to February 2017. Strict literature retrieval and data extraction were performed to extract relevant data. Data analysis was conducted using RevMan 5.3 software and Stata version 12.0. And relative risks (RRs) for survival rate were calculated. A fixed-effect model was selected to pool and a forest plot was used to display RRs.

**Results:**

Four studies involving 394 animals were finally included. Nine control groups are used to pool. A fixed-effect model was applied to estimate a pooled RR of 2.19 (95% CI: 1.74–2.76), indicating that PD-1 related blockade increased survival rate in sepsis animals.

**Conclusion:**

We concluded that PD-1 related blockade can improve survival of animals with sepsis. But robust standardized clinical experiments for sepsis patients are highly desirable.

## 1. Introduction

Sepsis is a complication caused by a disorder of host response to infection. Septic shock is a serious disease characterized by circulatory and cellular/metabolic dysfunction which is associated with a higher risk of mortality [1]. Not only is sepsis an important health and economic issue worldwide, but it is also a condition that is associated with morbidity and mortality in many hospitals. Moreover, the quality of life for the survivors of sepsis is impaired [2]. Over the past few decades, although antibiotics and fluid resuscitation have been used to counter sepsis widely, it remains the third most common disease that results in death in the United States [3] and there is an urgent need to develop novel therapies to treat sepsis [4]. Evidence from previous studies has indicated that, after the initial proinflammatory phase, sepsis is assumed to be severe immunosuppression, which is an important cause of deterioration in patients [5]. Several immunopathologic mechanisms have been reported to be involved in sepsis-induced immune alterations affecting both innate and adaptive immunities [6]. Therefore, inhibiting these immunopathologic alterations is widely considered a key step for the treatment of sepsis.

Programmed cell death-1 (PD-1) is expressed on activated T cells, natural killer cells, and B cells [7]. Programmed death-ligand 1 (PD-L1) is broadly expressed on hematopoietic and nonhematopoietic cells [8]. The expression of PD-1 on T cells and that of PD-L1 on monocytes were increased in patients with septic shock, and the PD-1/PD-L1 pathway might play an important role in sepsis-induced immunosuppression [9]. The PD-1/PD-L1 pathway inhibits T-cell activation, tolerance, and immunopathology. Moreover, Andriani et al. [10] have reported that defects in the immune function of patients with sepsis are associated with high PD-1 or PD-L1 expression and can be restored by treatment with antibodies targeting PD-1 or PD-L1.

Although several studies have explored the association between PD-1/PD-L1 expression and survival rate of patients with sepsis, few studies have explored the relationship between PD-1-related blockade and the survival rate of patients with sepsis because of medical ethics guidelines. Thus far, research of the effects of PD-1/PD-L1 blockade on sepsis survival has been limited to animal studies. In addition, although most previous reports focused on the effects of PD-1-related blockade in increasing the survival in animals with sepsis, there was no meta-analysis describing their effects. Thus, we performed a meta-analysis to assess the association between PD-1-related blockade and the survival rate in animals with sepsis. Our study aimed to shed new light on precise effects of the estimated treatment on the human trials, reduce the risk of false results [11], and provide a better understanding of the anti-PD-1/PD-L1 system.

## 2. Methods

### 2.1. Literature Search

To identify relevant studies published up to February 2017, we searched PubMed, EMBASE, and the Cochrane Library. The following combination of terms (“programmed cell death 1 blockade” or “PD-1 blockade” or “anti PD-1” or “programmed cell death-1 ligand blockade” or “anti PD- L1”) AND (“sepsis” or “septic shock” or “pyemia” or “septicemia”) were used.

### 2.2. Inclusion and Exclusion Criteria

The inclusion and exclusion criteria were designed based on the Cochrane handbook for systematic review of interventions (Version 5.3). The inclusion criteria were as follows: (1) evaluation of the association between PD-1 related blockade and survival rate in animals with sepsis; (2) independent randomized-controlled studies; (3) availability of full paper or acquiring it from the author; (4) selecting the most recent studies from the same author or institution; (5) manuscripts that were published in English.

We excluded studies if (1) enough data for pooling or additional data by contacting authors twice were not obtained; (2) the manuscripts comprised case reports, reviews, comments, abstracts, and editorials and clinical trials; (3) they were duplicate publications.

### 2.3. Data Collection Process

(1) Two reviewers (ZQ and QZJ) independently extracted relevant data, including study and animal features and outcomes from the key words, titles, abstracts, and full articles. When necessary, they compared the results, arrived at the same opinions, and solved disagreements by discussion with a third reviewer (LCS).

(2) Data pertaining to the dose and time of drug administration, animal age, country, and year of publication were used, including the number of cases in each group, results of the study, and related outcome, were extracted. If the survival outcomes were presented from the studies on animals at different time points, data for the last time point prior to the end of experiment were extracted

### 2.4. Quality Assessment

Due to the lack of tools for assessing the quality of randomized controlled trial (RCT) animal experiments, Peters' [12] system/meta-analysis report quality standards and literature evaluation sheet [13] were adopted. Based on CAMARADES (Collaborative Approach to Meta-Analysis and Review of Animal Data from Experimental Stroke), the lists were revised.

Study quality was evaluated for each publication using a modified 10-point checklist (Amarasingh et al., 2009) with one point allocated to each reported item: (1) peer reviewed publication, (2) sample size calculation, (3) random allocation to groups, (4) blinded assessment of outcome, (5) compliance with animal welfare regulations, (6) statement of potential conflicts of interest, (7) statement of control of temperature, (8) blinded application of PD-1 related blockade, (9) reported number of animals in whom the xenograft did not grow, and (10) presentation of evidence that acts directly against PD-1 related blockade.

### 2.5. Statistical Analysis

Review Manager 5.3 software was used to analyze data. We pooled data using relative risks (RRs) for continuous outcomes with corresponding 95% confidence intervals (95% CIs) to compare the differences. A fixed-effect model was selected if I^2^ was ≤ 50%. A random-effects model was selected if I^2^ > 50%. We performed subgroup analysis to identify the sources of heterogeneity. A sensitivity analysis was performed using Stata version 12.0 to identify influence of an individual study on the pooled RR. Publication bias was assessed using a funnel plot and Egger's test.

## 3. Results

### 3.1. Study Selection

**Figure 1:**
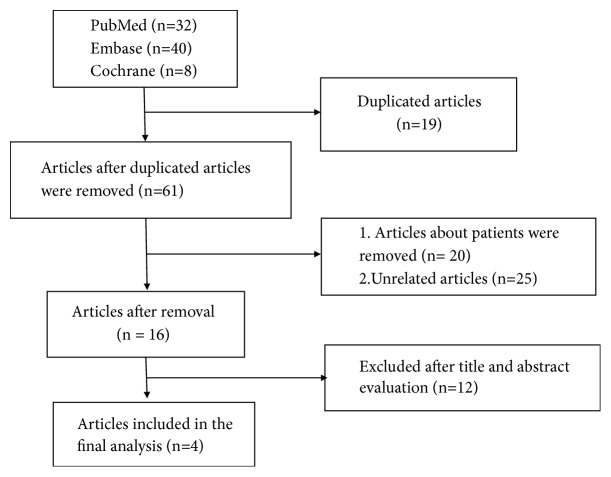
The flow of article selection.

A total of 80 studies were retrieved. Among these studies, 19 were duplicated and 57 of them were found to be unrelated to our study. Therefore, 4 studies [14–17] involving 394 mice were finally included in this study. The flow of article selection is shown in Figure 1.

Characteristics of the included studies are described in Table 1. All included studies used mice as the experimental animals and were published between 2010 and 2017. All the included studies used the cecal ligation and puncture (CLP) model of sepsis, and anti-PD-1 or anti-PD-L1 treatment was administered to mice at different time points. Zhang et al. [17] studied the effects of anti-PD-L1 by administration of the drug at two different time points, and isotype group and saline groups were selected as the control. In Brahmamdam's study [15], the experimental group was compared with the isotype and saline groups. Chang et al. [14] studied the effects of anti-PD-1 and anti-PD-L1 in sepsis models. Shindo et al. [16] and Zhang et al. [17] developed two-hit sepsis model of CLP because it reflected the impaired immune status of patients. Shindo et al. [16] explored the effects of anti-PD-L1 peptide (compound 8). Overall, nine groups were included in our analysis.

### 3.2. Quality Assessment

The overviews of the quality assessments of the four studies are shown in Table 2.

### 3.3. Meta-Analysis

**Figure 2:**
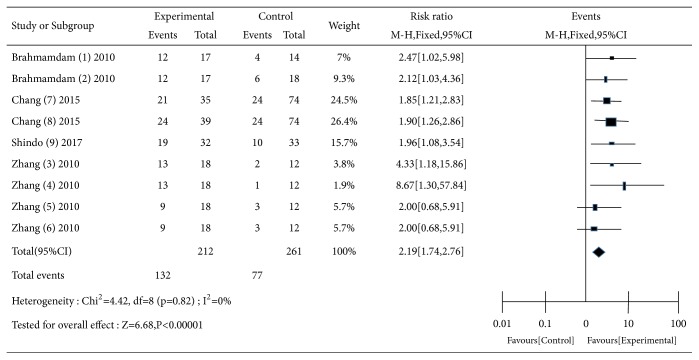
Forest of pooling effects of PD-1 related blockade in mice with sepsis.

All the included studies were used to estimate the effects of PD-1 related blockade on survival of animals with sepsis (Figure 2). There was no heterogeneity across the studies (Q = 4.42, df = 8, P = 0.82, and I^2^ = 0.0%). A fixed-effect model was applied to estimate a pooled RR. The result showed that blockade of PD-1/PD-L1 was associated with high survival rate (RR: 2.19; 95% CI: 1.74–2.76) in animals with sepsis.

**Figure 3:**
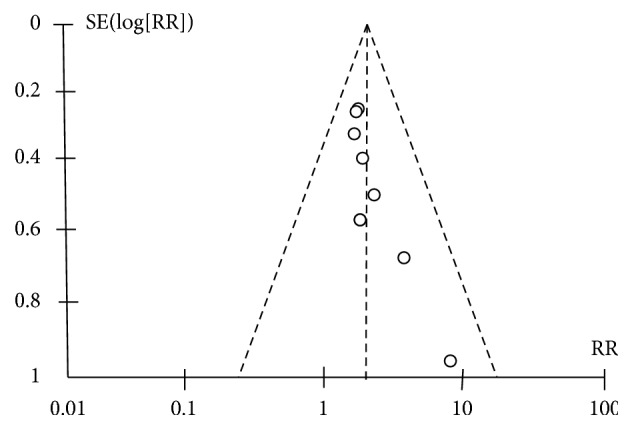
A funnel plot of effects of PD-1 related blockade in mice with sepsis.

A funnel plot (Figure 3) and Egger's test were used to assess the publication bias towards all the included studies. The funnel plot was unasymmetric, which demonstrated that there was no publication bias. Egger's test revealed no evidence of significant publication bias (P = 0.023).

**Figure 4:**
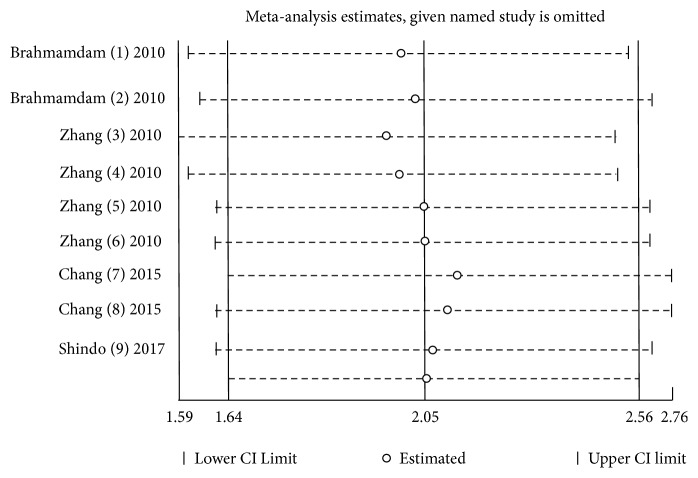
A sensitivity analysis of effects of PD-1 related blockade in mice with sepsis.

A sensitivity analysis (Figure 4)) was performed by excluding a single study each time. However, it did not change the outcome statistically.

Subgroup analysis (Table 3) was explored according to dose, drug, and administration time, but there was no difference between subgroups.

## 4. Discussion

Meta-analysis offers more significant evidence for clinical decision. Thus, this meta-analysis was conducted to assess the effects of PD-1 related blockade on survival in animal models of sepsis. Our major finding was that both anti-PD-1 and anti-PD-L1 have an increasing effect on the survival of mice with sepsis. This finding is based on a comprehensive systematic review, which included studies of over 394 mice. Moreover, because of medical ethics and other therapeutic methods, human studies remain difficult to conduct. Thus our results provide a good basis for further clinical research.

The variability in rats is far lower than that in patients. Furthermore, due to the strictly controlled experimental conditions, the results obtained in animal research are highly homogeneous, and the experimental procedures are highly repeatable. This may be the reason for the lack of heterogeneity in our meta-analysis.

PD-1 and its ligands PD- L1 are expressed not only on activated immune cells but also on several nonimmune tissues. Nishimura et al. [18] and Waterhouse et al. [19] found that deficiency of negative regulatory molecules results in severe hyperinflammatory and autoimmune diseases. Huang et al. found that PD-1 not only may be a marker of the developing of macrophages/monocytes dysfunction during sepsis but may also be a potential therapeutic target for designing measures to modulate the innate immune response, thereby preventing the detrimental effects of sepsis [20]. Shao et al. [21] found that PD-L1 expression on monocyte after 3–4 days of sepsis is associated with risk stratification and mortality in patients with sepsis. Identification of the importance of the immunosuppressive phase of sepsis has resulted in studies on various immune-adjuvants that could boost host immunity and improve the outcome [22]. Anti-PD-1 and anti-PD-L1 have led to a remarkable improvement in survival of patients with cancer [23, 24], a disease which shares several immunosuppressive mechanisms with sepsis. This prompted researchers to evaluate the effect of the blockade of the PD-1/PD-L1 pathway on sepsis [25]. Similarly, severe sepsis is associated with enhanced expression of the negative regulatory molecules, suggesting a novel approach to reverse immunoparalysis in sepsis. Brahmamdam et al. [15] reported that anti-PD-1 antibody could prevent the reduction of lymphocytes and dendritic cells in a mouse model of sepsis. Zhang et al. [17] showed that administration of anti-PD-L1 antibodies in mice suffering from sepsis could reduce the apoptosis of lymphocytes. Chang et al. [14] detected IFN-*γ* production and MHC II expression after anti-PD-1 administration and found that PD-1/PD-L1 blockade can improve immune cell functions. Overall, PD-1/PD-L1 pathway may play key roles in triggering the immunosuppression in patients with sepsis, and PD-1/PD-L1 blockade might promote the recovery of immune cell functions and increase ability of microbial clearance. Consequently, we concluded that blockade of PD-1/PD-L1 appears to improve survival in animal models of sepsis. However, robust standardized clinical experiments on patients with sepsis are highly desirable.

We could not include studies, such as that of Bergerat [26], who found that anti PD-L1 treatment 6h after CLP did not improve survival in mouse model, as it was a conference abstract. Among the included studies in our study, Zhang et al. [17] concluded that anti PD-L1treatment 3h after CLP could improve survival in a mouse model. The present study demonstrated that, among PD-1-related molecules, only PD-L1 expression on monocyte after 3–4 days of sepsis was valuable for the risk stratification of patients with sepsis [21]. This may be because the immunosuppression phase is characterized at days 3–4 after the onset of sepsis. Thus, several clinical studies on the role of early blockade of PD-1-related molecules (at days 1–2) in improving survival of patients with sepsis are urgently needed.

### 4.1. Sample Size

The preclinical experiment should include enough data to detect a treatment effect if such an effect truly exists. However, studies included in our analysis were of inadequate size. Trials with small sizes often have the risk of overestimation of intervention benefits [27]. Overestimated studies are much less prevalent [28]. In addition, the sample size should be calculated before the initiation of the study with a formal calculation, of which the fundamental elements of statistical significance, such as effect size, power, and standard deviation of the measurements, have been made clear in numerous articles [29]. In addition, PD-1/PD-L1 blockade therapy-associated adverse events, such as diarrhea, pneumonitis, and diabetes, should be considered [30].

### 4.2. Strengths and Limitations of This Meta-Analysis

In recent years, increasing studies such as those of Liu et al. [31] and Sherwood et al. [4] have demonstrated that PD-1/PD-L1 blockade could be used to treat sepsis. However, their studies focused on the mechanism of action. There are several strengths of this meta-analysis. First, our meta-analysis was performed according to the recommendations given in Preferred Reporting Items for Systematic Reviews and Meta-Analyses (PRISMA) statement protocol [32]. Three electronic databases were searched according to the Cochrane Collaboration. The screening of eligible studies, assessment of methodological quality, and data extraction were conducted independently. Second, we included only RCTs in our review to minimize potential bias and a large number of studies were used to address this question. Meanwhile, there was no significant heterogeneity among the included studies. Third, several subgroup analyses were explored according to dose, drug, and administration time, and sensitivity analyses were also performed to verify the robustness of our results.

There were some limitations in our study: (1) our sample size does not have sufficient statistical power; (2) after the initial screening and subsequent application of exclusion criteria, only four articles were included for the meta-analysis, although we searched several databases. This, in turn, might not allow for us to draw reliable conclusions; (3) the methodological quality of the included studies was generally low, which is an inherent limitation. This may result in an overestimation of effect size [33]; (4) information pertaining to the environment in which the mice were treated was not described, making it impossible to assess the effect of this potentially important factor with regard to implementing a control; (5) we also did not explore more specific mechanisms underlying the protective role of the PD -1-related blockade in mice with sepsis; (6) the included studies showed different treatment time window of the blockade of PD-1/PD-L1 pathway, which may be better for good experimental results but may be difficult to be some indications for clinical research. However, to our knowledge, this is the first meta-analysis to estimate the effects of the PD-1-related blockade in sepsis, which might be informative for future clinical experiments.

## 5. Conclusion

Blockade of PD-1/PD-L1 appears to improve the survival in animals with sepsis. Large randomized clinical trials testing the effect of PD-1/PD-L1 blockade in patients with sepsis are warranted.

## Figures and Tables

**Table 1:** Characteristics of the included studies.

Paper/author	Animal/sex	Drug	Country	Administration time	Dose	Age	Year	Control group

Brahmamdam et al.	Mice/male	Anti-PD-1	USA	24-48h after CLP	200ug in 200mL saline	8-10w	2010	(1)isotype
								(2)saline
Zhang et al.	Mice/male	Anti-PD-L1	CHINA	(a) 24h before CLP	50 *μ*g/mouse	8-10w-	2010	(3)isotype
								(4)saline
				(b) 3h after CLP				(5)isotype
								(6)saline
Chang et al.	Mice/male	(a) Anti-PD-1	USA	24-48h after CLP	200ug-	8-10w	2015	(7)isotype
		(b) Anti-PD-L1					2015	(8)isotype
Shindo et al.	Mice/male	Anti-PD-L1	USA	48h after CLP	-* *-	6-8w	2017	(9)inactive

**Table 2:** Quality assessment of included studies.

Study/author	1	2	3	4	5	6	7	8	9	10	score
Brahmamdam et al.	+	-	+	-	+	-	-	-	-	-	3
Zhang et al.	+	-	-	-	+	+	-	-	-	-	3
Chang et al.	+	-	-	-	+	-	-	-	-	-	2
Shindo et al.	+	-	-	-	+	+	-	-	-	-	3

**Table 3:** Subgroup results of PD-1 related blockade on survival in animals with sepsis.

Subgroup basis	Group type	Included studies (n)	p-value	RR (95%CI)	I^2^	Subgroup difference
Dose	200 ug	4	0.94	1.97 (1.51-2.56)	0	P=0.14
	50 ug	4	0.43	3.26 (1.75-6.08)	0	
Drug	Anti-PD-1	3	0.83	2.02 (1.43-2.82)	0	P=0.56
	Anti-PD-L1	6	0.54	2.31 (1.70-3.14)	0	
Administration time	<48h	8	0.73	2.23 (1.74-2.81)	0	P=0.69
	>48h	1	0.03	1.96 (1.08-3.54)	0	

## Data Availability

All data during the current study are available for consultation and for the public, upon request to the corresponding author.
